# One Lipid, Two Synaptic Plasticity Pathways

**DOI:** 10.1371/journal.pbio.1002154

**Published:** 2015-05-21

**Authors:** Richard Robinson

**Affiliations:** Freelance Science Writer, Sherborn, Massachusetts, United States of America

## Abstract

A new study identifies an unexpected role for lysophosphatidic acid in modulating the strength of both excitatory and inhibitory synapses in the brain, but by different mechanisms. Read the accompanying Research Article.

The strength of a synaptic connection—that is, the likelihood that a presynaptic stimulus will lead to a post-synaptic response—can change from moment to moment, as experience, the local cellular environment, and the neuron’s own need for homeostasis are combined and minute molecular adjustments are made to axon and dendrite.

Understanding the molecular mechanisms underlying synaptic plasticity is a major goal of current neuroscience research. In this issue of *PLOS Biology*, Victoria García-Morales, Bernardo Moreno-López, and colleagues show that a phospholipid plays a key role in fine-tuning both excitatory and inhibitory synapses. Remarkably, the phospholipid sets in motion two entirely different signaling cascades in the two types of synapses, and it exerts its final effects through two entirely different mechanisms.

The phospholipid is lysophosphatidic acid (LPA), which is derived from membrane lipids and is known to act as a signaling molecule, especially in the brain, where its receptors are highly expressed. That fact led the authors to explore whether LPA might play a role in modulating synaptic plasticity.

Working in rodents and cultured neurons, they began by demonstrating that adding LPA at physiologic doses to excitatory neurons (those that use glutamate as a neurotransmitter) reduced the amplitude of the output current of the post-synaptic neuron, a phenomenon called short-term depression (STD). Elsewhere, LPA is known to signal through a downstream G protein, and adding an inhibitor of that protein prevented LPA’s ability to induce STD. Immunolabeling of the LPA receptor showed that it was active in the pre-synaptic neuron, where it colocalized with a marker of neurotransmitter-containing vesicles, suggesting it might exert its effect by inhibiting neurotransmitter release (Fig 1).

**Fig 1:**
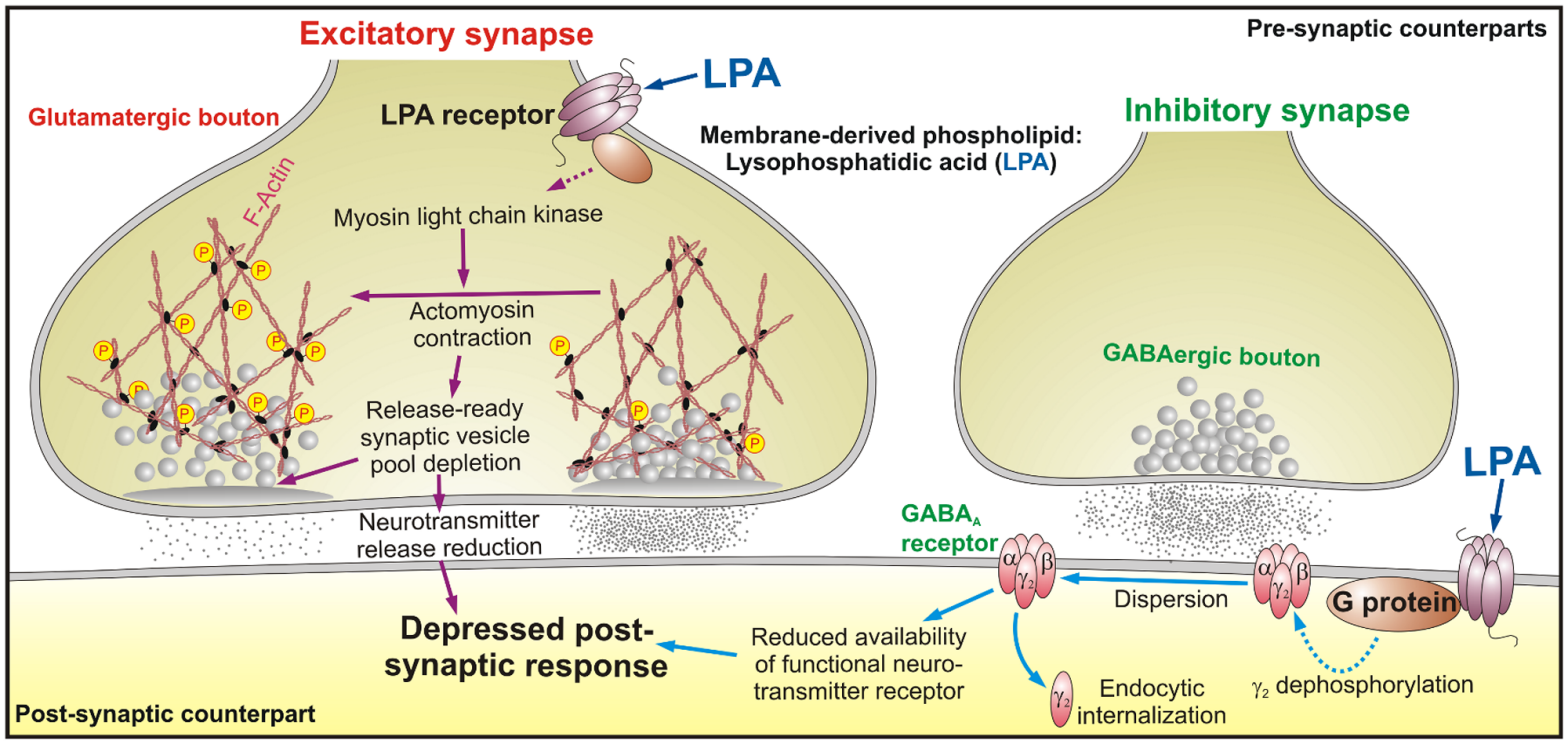
Membrane-derived phospholipids, such as lysophosphatidic acid (LPA), an important intermediary in lipid metabolism, are important determinants of neuron activity by regulating synaptic function. LPA induces short-term depression at both excitatory and inhibitory synapses by recruiting different signaling cascades. Because membrane-derived phospholipids, such as LPA, are intermediaries in lipids’ metabolism, these results suggest that bioactive phospholipids are potential candidates in coupling the metabolic status of the organism to brain function. *Image credit*: *Bernardo Moreno-López*.

As part of the neuronal cytoskeleton, as well as through its interaction with actin, myosin light chain helps control the movement of neurotransmitter-containing vesicles. In smooth muscle, LPA stimulates the enzyme myosin light chain kinase (MLCK) to add a phosphate to the myosin light chain, activating it and promoting actomyosin contraction. The authors found that an inhibitor of MLCK prevented LPA-induced STD, and under the electron microscope, it became clear why: LPA, through its regulation of MLCK, was triggering actomyosin to contract, and in so doing withhold vesicles from the plasma membrane, thereby reducing the release of neurotransmitter and causing STD in the post-synaptic neuron.

At inhibitory neurons, which use GABA as a neurotransmitter, they also found that addition of LPA induced STD, but through an entirely different mechanism. Here, LPA acted through receptors on the post-synaptic neuron, which colocalized with a marker for a protein that helps assemble GABA receptors from its subunits. LPA exerted its effect through the RhoA/ROCK signaling pathway, at the downstream end of which was calcineurin, a protein known to dephosphorylate certain GABA subunits. Addition of LPA reduced the proportion of phosphorylated GABA subunits among the total GABA subunit pool, which previous work has shown is associated with dispersion of the subunits and inactivation of the receptor, thereby reducing GABA signaling and inducing STD.

Finally, the authors showed that, in vivo, LPA signaling was crucial for proper regulation of motor neuron output. They found that application of LPA inhibitors to rhythmically firing neurons prevented them from maintaining a steady output rhythm in response to increased excitation.

This final result suggests that one role of LPA-induced STD is to maintain “the brakes” on motor output in the face of increased sensory input. Among other benefits, such a system of restraint may prevent excitotoxicity, in which excessive firing hastens neuronal death. More broadly, the system, in combination with others, allows fine-tuning of a synapse’s behavior over a very short time span.

On an even wider scale, the fact that a lipid functions as a synaptic regulator may provide a mechanistic link between metabolic syndromes and neurologic diseases, the authors suggest, and have important implications for understanding how an organism’s metabolic state and brain function are coupled.
